# Emerging role of F-box proteins in the regulation of epithelial-mesenchymal transition and stem cells in human cancers

**DOI:** 10.1186/s13287-019-1222-0

**Published:** 2019-04-18

**Authors:** Yizuo Song, Min Lin, Yi Liu, Zhi-Wei Wang, Xueqiong Zhu

**Affiliations:** 1 Department of Obstetrics and Gynecology, The Second Affiliated Hospital of Wenzhou Medical University, No. 109 Xueyuan Xi Road, Wenzhou, 325027 Zhejiang China; 2 Center of Scientific Research, The Second Affiliated Hospital of Wenzhou Medical University, Wenzhou, 325027 Zhejiang China; 3 Department of Pathology, Beth Israel Deaconess Medical Center, Harvard Medical School, Boston, MA USA

**Keywords:** EMT, F-box protein, Cancer, Stem cells, Drug resistance, Metastasis

## Abstract

Emerging evidence shows that epithelial-mesenchymal transition (EMT) plays a crucial role in tumor invasion, metastasis, cancer stem cells, and drug resistance. Data obtained thus far have revealed that F-box proteins are critically involved in the regulation of the EMT process and stem cell differentiation in human cancers. In this review, we will briefly describe the role of EMT and stem cells in cell metastasis and drug resistance. We will also highlight how numerous F-box proteins govern the EMT process and stem cell survival by controlling their downstream targets. Additionally, we will discuss whether F-box proteins involved in drug resistance are associated with EMT and cancer stem cells. Targeting these F-box proteins might be a potential therapeutic strategy to reverse EMT and inhibit cancer stem cells and thus overcome drug resistance in human cancers.

## Background

Epithelial-mesenchymal transition (EMT) is a molecular reprogramming cellular process that is characterized by transition of polarized immotile epithelial cells to motile mesenchymal cells and that involves phenotypic changes [1]. This process is required for tissue remodeling during embryonic development [2]. Subsequently, it has been reported that EMT contributes pathologically to cancer progression, as tumor cells exhibit increased migratory and invasive abilities [2]. During this transition, the expression of epithelial proteins that enhance cell-cell adhesion such as E-cadherin and γ-catenin is decreased, while the expression of mesenchymal markers such as vimentin, N-cadherin, and fibronectin as well as the activity of some matrix metalloproteinases (MMPs) are increased [3]. Furthermore, cancer cells are able to obtain cancer stem cell (CSC) features through induction of EMT [4], which has become a major cause of tumor relapse and metastasis [5] and results in increased resistance to chemo- and immunotherapies.

EMT has been found to be induced by multiple signaling regulators, including Snail, Twist, and zinc-finger E-box-binding (ZEB) transcription factors [6, 7]. As these transcription factors have distinct expression profiles, their contributions to EMT depend on the cancer cell type or specific tumor tissues involved [8]. Moreover, many oncogenic signaling pathways trigger the initiation and progression of EMT. For example, transforming growth factor-β (TGF-β) signaling is the most well-characterized pathway that promotes EMT in a variety of human cancer cells [9]. Similarly, Wnt, Akt, Hedgehog, Notch, RTK, matrix metalloproteinases, hypoxia, and nuclear factor-κB (NF-κB) have also been confirmed to induce EMT [2, 10, 11]. Additionally, many differential expression studies of microRNA (miRNA) have been performed to identify candidate miRNAs that possibly regulate EMT [12]. Specifically, these noncoding miRNAs could selectively bind to mRNAs and subsequently inhibit their translation or facilitate their degradation, thus controlling the expression of EMT master transcription factors [13]. More importantly, F-box proteins have been well studied and have been demonstrated to be associated with tumorigenesis, and some of them are relevant in the regulation of EMT [14, 15].

Stem cells exhibit an unlimited capacity for self-renewal and the potential to differentiate into different cell types, and thus, they can subsequently form tissues and organisms [16]. Three types of stem cells have been identified: embryonic, germinal, and somatic. In recent years, cancer stem cells (CSCs), which are also known as cancer stem-like cells, have been validated to exist in various types of human cancers, although the concept of CSCs remains controversial [17]. Multiple factors such as Notch, Wnt, and Sonic hedgehog have been reported to trigger the process of stem cell differentiation. Recently, accumulating evidence has demonstrated that F-box proteins play an important role in the regulation of CSCs. Therefore, in this review, we will primarily describe emerging F-box proteins that are involved in the regulation of EMT and CSCs in human cancers.

## The ubiquitin-proteasome system (UPS) and the classification of E3 ubiquitin ligases

Protein degradation is often essential for a rapid response to signal transduction and the recycling of amino acids as part of protein turnover, and a dysregulated pool of proteins may lead to various types of disorders including cancer [18, 19]. Studies have demonstrated that two major proteolytic pathways function in eukaryotes, namely, lysosomal-mediated and proteasome-mediated degradation proteolysis [20, 21]. The ubiquitin-proteasome system (UPS) regulates cellular protein homeostasis by governing the process of protein degradation, which is known as ubiquitination, and subsequently controls different cellular processes such as cell proliferation, cell cycle progression, migration, and apoptosis [18, 22, 23]. Some specific molecular signaling pathways would be constantly activated by damaged or unwanted proteins that were not removed in a timely manner, which could cause diseases such as cancer [24].

It is known that UPS-targeted protein degradation involves two well-defined steps: (1) the covalent attachment of multiple ubiquitin molecules to a substrate and (2) the recognition and degradation of a ubiquitin-tagged substrate by the 26S proteasome [25, 26]. The first step occurs by a three-step enzymatic cascade, which is catalyzed by the ubiquitin-activating enzyme (E1), the ubiquitin-conjugating enzyme (E2), and the ubiquitin ligase (E3) [14]. Specifically, the ubiquitin molecule is activated by E1 in an ATP-dependent manner and is transferred to E2. Subsequently, E3 promotes the attachment of ubiquitin moieties to the substrate protein, which finally facilitates degradation by the 26S proteasome [27].

It is noteworthy that the substrate specificity of ubiquitination and further degradation is determined by the E3 enzymes [28]. As of now, more than 600 E3 ubiquitin ligases have been discovered in the human genome [29]. E3 ligases are mainly divided into three major types: U-box domain, really interesting new gene (RING) domain, and homologous to E6-associated protein C-terminus (HECT) domain [30]. In addition, the largest family of E3s is the Cullin-RING E3 ligase (CRL) complex family, which contains eight members named CRL1, CRL2, CRL3, CRL4A, CRL4B, CRL5, CRL7, and CRL9 [31, 32]. Of the eight CRLs, CRL1, also designated as the SKP1-cullin 1-F-box protein (SCF) E3 ligase complex, has been well characterized [33]. Structurally, the SCF complex consists of CRL1 as the scaffold protein; the RING finger protein RBX1, which recruits the E2 enzyme; and S-phase kinase-associated protein 1 (SKP1); SKP1 functions as an adaptor protein to bridge F-box proteins as well as variable F-box proteins that confer substrate selectivity by targeting a distinct subset of substrates for ubiquitination [34, 35]. It has been validated that the human genome encodes 69 F-box proteins, and each of them is composed of at least two functional domains: various carboxy-terminal domains that bind specific substrates [35] and the F-box motif, which is considered to be a protein-protein interaction domain that recruits F-box proteins into the SCF complex via direct binding with the adaptor protein SKP1 [36]. Depending on the specific substrate recognition and binding domains, F-box proteins are organized into three subclasses: 10 FBXW proteins (contain WD40 repeat domains), 22 FBXL proteins (contain leucine-rich repeat substrate binding domains), and 37 FBXO proteins (contain other motifs such as kelch repeats or proline-rich motifs to bind substrates) [34, 37]. Therefore, this article will provide a comprehensive summary of the known and emerging F-box proteins that regulate EMT and CSCs and will elucidate how F-box proteins, by targeting their substrates, are involved in EMT and CSC regulation during carcinogenesis.

## Regulation of EMT by F-box proteins

Numerous efforts have been made to identify several F-box proteins that are involved in EMT through degradation of their downstream proteins. However, it is worth mentioning that studies on F-box proteins and EMT are still scarce and that data are lacking for most of the 69 F-box proteins. In the following sections, we will describe the specific substrates, functions, and pathological evidence of different F-box proteins that are involved in EMT regulation (Table 1).

**Table 1:** Substrates, functions, and pathological evidence of F-box proteins involved in EMT during cancer progression

F-box protein	Substrates	Cancer types	Functions	References
β-TrCP	Snail, β-catenin, Twist	Breast cancer, lung cancer, nasopharyngeal carcinoma, and cervical cancer	Oncoprotein or tumor suppressor; inhibits EMT, migration, and invasion	[63, 64, 71–75]
FBXW7	Snail, MMPs, mTOR, RhoA	Lung cancer, gastric cancer, renal cell carcinoma, colorectal cancer, liver cancer, and cholangiocarcinoma	Tumor suppressor; inhibits EMT, invasion, and metastasis; and increases sensitivity to chemotherapeutic drugs	[39–44, 50–52, 136]
SKP2	p27, Twist	Melanoma, breast cancer, osteosarcoma, and prostate cancer	Oncoprotein; promotes cell growth, EMT, and invasion; and increases drug resistance	[84, 86, 87, 139]
FBXL5	Snail1	Gastric cancer and cervical cancer	Tumor suppressor; inhibits EMT and metastasis	[92, 94, 95]
FBXL14	Snail1, Slug, Twist, Sip1	Head and neck cancer	Tumor suppressor; inhibits EMT and metastasis	[97, 98]
FBXO2	None	Gastric cancer	Oncoprotein; promotes proliferation, metastasis, and EMT	[127]
FBXO11	Snail	Breast cancer and gastric cancer	Tumor suppressor; inhibits cell growth, EMT, and metastasis; induces apoptosis	[102–104]
FBXO22	Snail	Breast cancer	Tumor suppressor; inhibits EMT and metastasis	[131]
FBXO31	Snail1	Gastric cancer	Tumor suppressor; inhibits cell growth and EMT	[135]
FBXO32	MyoD, CtBP1	Urothelial carcinoma and breast cancer	Induces apoptosis and inhibits EMT in urothelial carcinoma; promotes EMT and metastasis in breast cancer cells	[113, 114]
FBXO45	Snail1, Snail2, Twist1, ZEB1, ZEB2	Prostate cancer, breast cancer, lung cancer, liver cancer, cervical cancer, and pancreatic cancer	Tumor suppressor or oncoprotein; inhibits EMT	[122–124]

### The FBXW family

Two types of FBXW proteins have been found to regulate the EMT process in different types of human cancers, including the well-studied FBXW7 (also known as FBW7 and CDC4) and β-transducin repeat-containing protein (β-TrCP). FBXW7 plays an important tumor-suppressive role in regulating the effects of various oncogenic proteins [37], including cyclin E, c-Myc, c-Jun, Notch, mTOR, and MCL-1 [37, 38]. The transcription factor Snail, a key regulator of EMT, has recently been found to be regulated by FBXW7 [39]. In this study, FBXW7 suppressed cell migration and invasion mainly by promoting ubiquitination and proteolysis of Snail in human nonsmall cell lung cancer (NSCLC), which led to inhibition of E-cadherin expression in vitro and in vivo. Moreover, silencing of FBXW7 induced EMT through the stabilization of Snail protein, which indicates that the FBXW7-Snail axis could be a potential target for anti-NSCLC therapy [39]. Furthermore, another study revealed that FBXW7 inhibited the metastasis and progression of gastric cancer partly via the RhoA signaling pathway, which was accompanied by upregulation of E-cadherin and downregulation of N-cadherin and vimentin [40]. A mechanistic study demonstrated that FBXW7 induced ubiquitination and proteasomal degradation of RhoA protein, which led to a blockade of EMT [40].

It was recently reported that FBXW7 is frequently downregulated in cancer cell lines and tumor tissues, such as renal cell carcinoma (RCC), compared with normal tissues and normal cell lines [41]. Functional studies revealed that the increase in FBXW7 levels dramatically impedes the migration and invasiveness of RCC cells through a decrease in MMP expression, which suggests that FBXW7 exhibits notable anticancer properties in RCC via repression of EMT [42, 43]. Additionally, rapamycin, which is an mTOR inhibitor, attenuates the EMT process and stem-like properties that are driven by FBXW7 loss, which indicates the pivotal role of the FBXW7/mTOR axis in EMT regulation in colorectal cancer [44]. An integrated analysis of the Cancer Genome Atlas (TCGA) network identified several frequent mutations, including ones in FBXW7, that were associated with EMT features in uterine carcinosarcomas [45]. In line with this, FBXW7 mutations were found in colorectal carcinomas, T-ALL, cholangiocarcinoma, uterine endometrial carcinoma, and bladder carcinoma [46]. Similarly, Sakai et al. reported that combined mutations in FBXW7^−/−^ and Kras^G12D^ induced EMT-like morphology in mucosal tumors in an intestinal cancer model [47]. In fact, mutations in FBXW7 may impair its ability to bind to its substrates; this would lead to upregulation of the active forms of these substrates such as Notch1 [48] and c-Myc [49], which are involved in EMT and cancer progression.

Increasing evidence shows that the FBXW7 protein could overcome EMT-induced resistance to chemotherapy in malignant tumors [50]. For instance, one group recommended that an increase in FBXW7 apparently enhanced cisplatin chemosensitivity in NSCLC cells [51]. Likewise, FBXW7 was also reported to improve the cytotoxicity of doxorubicin and to ablate the invasive capability of hepatocellular carcinoma (HCC) cells via inhibition of EMT [52]. Interestingly, Ding et al. found that overexpression of miR-223 could reduce the FBXW7 levels, which promoted EMT and doxorubicin resistance in colorectal cancer cells [53]. Moreover, one study indicated that miR-223 was able to induce EMT and decrease sensitivity to doxorubicin through targeting FBXW7 in NSCLC [54]. Furthermore, several investigations have found that FBXW7 is also a downstream target of miR-367 [55–57]. Interestingly, the novel long noncoding RNA (lncRNA) cancer susceptibility candidate 2 (CASC2) was reported to inhibit EMT and exert its antimetastatic effects via the miR-367/FBXW7 pathway in HCC cells [58]. Therefore, these findings define a crucial role of the miRNAs/FBXW7 axis in EMT regulation, which may facilitate the development of individualized treatment in clinical practice.

Other FBXW proteins, β-TrCP1 (also named FBXW1) and β-TrCP2 (also named FBXW11), exhibit their oncogenic or tumor-suppressive functions based on the specific cancer type or cellular context [14]. Twenty years ago, IκBα (the NF-κB inhibitor) and β-catenin were identified as two substrates of β-TrCP1 [59, 60]. Since then, other substrates of β-TrCP1 and the highly related β-TrCP2 have been identified [61]. Notably, β-TrCP1 and β-TrCP2 have indistinguishable biochemical functions in terms of recognizing and degrading their substrate proteins [62]. Previous studies have demonstrated that Snail is a labile protein that contains two phosphorylation motifs of glycogen synthase kinase-3β (GSK-3β), which are each necessary for protein degradation and subcellular localization [63]. GSK-3β binds and phosphorylates Snail at motif 2 and thereby induces its nuclear export [63]. Subsequent phosphorylation by GSK-3β at motif 1 results in the association of Snail with β-TrCP and thus leads to the ubiquitination and degradation of Snail in the cytoplasm [63]. Furthermore, cross talk among different signaling pathways including Wnt, hedgehog, and FGF induced EMT during carcinogenesis and were found to be controlled by GSK-3β and β-TrCP through degradation of β-catenin and Snail [64, 65]. It is important to note that missense mutations in β-TrCP such as A99V, H342Y, H425Y, C206Y, G260E, and F462S, which could lead to β-catenin stabilization, have been observed in gastric cancer [66, 67]. In addition, mutations in β-TrCP have also been reported in prostate and breast cancers [68, 69].

Notably, Epstein-Barr virus (EBV) infection plays a critical role in the carcinogenesis and development of nasopharyngeal carcinoma (NPC), as the virus could encode some miRNAs that facilitate tumor progression through targeting virus-infected host genes or self-viral genes [70]. In 2015, Yan et al. demonstrated that EBV-miR-BART10-3p promoted EMT and metastasis in NPC by directly targeting β-TrCP and stabilizing the level of the downstream substrates β-catenin and Snail [71]. In addition, one group revealed that NF-κB abrogated the ubiquitination and degradation of Snail by β-TrCP via the induction of COP9 signalosome 2 (CSN2) [72]. Western blot results also showed that the expression of Snail was related to the activation of NF-κB, while knockdown of Snail suppressed inflammation-mediated invasion and migration of breast cancer cells [72]. YWHAZ, also known as 14-3-3zeta, was identified as a potent EMT promoter in NSCLC. Moreover, the level of ubiquitinated β-catenin is decreased due to its interaction with YWHAZ, which results in detachment of β-catenin from β-TrCP and a subsequent increase in β-catenin stability [73]. Twist has been considered one of the key factors of EMT through its inhibition of E-cadherin expression, which leads to enhanced cell invasion and metastasis [7]. One study identified Twist protein as a ubiquitin substrate of β-TrCP and found that the degradation of Twist could inhibit EMT and metastasis of human cervical cancer [74]. Moreover, Li et al. reported that AKT1 suppressed EMT via induction of β-TrCP-mediated Twist1 degradation in breast cancer cells, which ablated cell migration and invasion [75]. Hence, considering the function of β-TrCP in regulating EMT, targeting β-TrCP could be a new therapeutic treatment for human cancers. In summary, FBXW7 and β-TrCP inhibit the EMT process via degradation of their substrates in human cancer cells.

### The FBXL family

The FBXL subfamily contains 22 members, namely, FBXL1 (also known as SKP2) to FBXL22. All the FBXL proteins contain an F-box motif and a C-terminal Leu-rich repeat (LRR) domain. Several FBXL subfamily member proteins have been widely reported to affect carcinogenesis and cancer progression. In the following section, we will describe the roles of FBXL proteins, including SKP2, FBXL5, and FBXL14 (also known as the F-box protein Ppa), in EMT regulation.

A growing body of literature strongly implies that SKP2 is an oncoprotein in human cancers [34]. Actually, SKP2 was first identified as a key cell cycle regulator because it targets multiple cell cycle-related proteins such as p27 [76, 77] and p21 [78, 79]. Several studies have confirmed that p27 is a primary target substrate of SKP2 [80, 81] and that its expression is inversely related to SKP2 expression in human cancers [82]. Furthermore, SKP2 plays important roles in cancer metastasis via decreasing the expression of p27 [78]. It has also been documented that TGF-β1 induces EMT [83]. Interestingly, one study revealed that the level of SKP2 was elevated by TGF-β1 treatment in human melanoma, which was accompanied by increased phosphorylation of Akt1 and accumulation of c-Myc during EMT [84]. One group showed that miR-200b/c induced the upregulation of SKP2 and the degradation of p27 via targeting the reversion-triggering cysteine-rich protein with Kazal motifs (RECK), which promoted tumorigenesis and progression in colorectal cancer [85].

Paclitaxel is an important drug used as a pivotal chemotherapeutic agent in the treatment of breast cancer. However, patients with breast cancer often develop drug resistance, which leads to poor treatment outcomes. One group reported that EMT was correlated with high expression of SKP2 and that knockdown of SKP2 caused partial reversal of the EMT phenotype in paclitaxel-resistant breast cancer cells [86]. Methotrexate (MTX)-resistant osteosarcoma, in which EMT is involved, remains a major clinical challenge and usually affects adolescents and young adults [87]. EMT properties and resistance to MTX in OS cells are acquired via SKP2 overexpression [87]. Thus, the pharmacological suppression of SKP2 may prove to be a potent chemotherapeutic strategy to overcome drug resistance due to EMT reversal in various human cancers. Several Skp2 inhibitors, such as Compound A and SZL-P1-41, have been discovered to block Skp2 E3 ligase activity [88, 89].

FBXL5 was found to regulate the turnover of p150, which acts as an activator of the microtubule motor cytoplasmic dynein; this protein is important for vesicular transport and mitotic spindle organization [90]. However, the role of FBXL5 in tumorigenesis has not been fully elucidated. It has been reported that FBXL5 could modulate Snail1 binding to DNA and its stability [91]. Moreover, Snail1 was validated as a substrate of FBXL5, and the degradation of Snail1 leads to the negative regulation of EMT, which inhibits the metastasis of gastric cancer cells [91, 92]. The protein iASPP, encoded by the PPP1R13L gene, is overexpressed in human cancers and increases the expression of miR-20a in a p53-dependent manner [93]. Furthermore, Xiong et al. reported that the iASPP-induced upregulation of miR-20a promoted the EMT process and led to cisplatin resistance partially through targeting FBXL5 in cervical cancer cells [94]. In contrast, one group observed that the silencing of FBXL5 was sufficient to upregulate E-cadherin at the plasma membrane, which is the basic structure of cell-cell adhesion [95]. Therefore, the functions of FBXL5 in the regulation of EMT should be further investigated in human cancers.

FBXL14, also known as the F-box protein Partner of paired (Ppa), is dynamically expressed in neural crest-forming regions [96]. Importantly, the expression of FBXL14 is abrogated in tumor tissues [96]. Additionally, it has been shown that the FBXL14 protein mediates proteasome degradation of core EMT factors, including Snail1, Twist, Sip1, and Slug [97]. Consistently, one group revealed that imipramine blue inhibited head and neck cancer cell invasiveness by enhancing FBXL14-triggered Twist degradation, which indicates that FBXL14 could function as an EMT inhibitor to suppress metastasis in human cancers [98]. Undoubtedly, further investigations are required to determine the physiological role and mechanisms of FBXL proteins in EMT regulation in various types of human cancers. In summary, SKP2 enhances EMT in breast cancer and melanoma, whereas FBXL5 suppresses EMT in human gastric and cervical cancer cells, and FBXL14 is an EMT inhibitor in head and neck cancer cells.

### The FBXO family

Except the FBXW and FBXL families, the remaining 37 F-box proteins are designated as FBXO proteins, which comprise the largest subclass of the 69 putative F-box proteins. Unlike FBXW proteins with the WD40 motif and FBXL proteins with the LRR motif, FBXO proteins contain the F-box motif in the N-terminus and different functional domains in the C-terminus, which have not been fully characterized. In this section, we will limit our discussion to several members of the FBXO family, which function in modulating EMT, including FBXO2, FBXO11, FBXO22, FBXO31, FBXO32, and FBXO45.

It has been reported that FBXO11 targets the BCL-6 oncogenic protein for ubiquitination and subsequent degradation, which is implicated in the pathogenesis of human B cell lymphomas [99]. Biologically, the overexpression of FBXO11 inhibits cell proliferation and induces cell apoptosis by facilitating BCL-6 degradation in diffuse large B cell lymphoma cells [99]. In addition, FBXO11 mutations were observed in patients with splenic marginal zone lymphoma, diffuse large B cell lymphomas, and pancreatic cancer [99–101]. It has been previously demonstrated that FBXO11 is a ubiquitin ligase that promotes ubiquitin-induced degradation of Snail through targeting the SNAG domain of Snail1, which suppresses the progression of EMT in breast cancer cells [102]. Another study using an animal model showed that deactivation of FBXO11 in mice resulted in neonatal lethality, epidermal thickening, and upregulation of Snail in the epidermis, which suggests that FBXO11 is a physiological ubiquitin ligase of Snail [102]. Likewise, Zheng et al. revealed that SCF-FBXO11 attenuated the EMT process and cancer metastasis by promoting the degradation of Snail in a PKD1 phosphorylation-dependent manner; this is considered posttranslational regulation of EMT [103]. Nevertheless, the expression of FBXO11 was found to be upregulated in gastric cancer tissues compared with tumor-adjacent tissues, while a clinical analysis based on the TCGA database indicated that the elevation in FBXO11 level was closely related to large tumor size, lymph node metastasis, and advanced TNM stage [104]. Mechanistically, the EMT process is dramatically stimulated by FBXO11 through the PI3K/Akt signaling pathway, which leads to the promotion of gastric cancer cell proliferation and metastasis [104].

FBXO32, which is also known as a muscle-specific F-box protein, is expressed largely in skeletal muscle cells and cardiomyocytes [105, 106]. It has been documented that FBXO32 plays a crucial role in regulating muscle homeostasis by targeting multiple substrates such as calcineurin [107], eIF-3 [108], MyoD [109], MAPK-1 [110], and IκBα [111]. Emerging evidence has identified FBXO32 as a tumor suppressor because it induces apoptosis [112]. In support of this evidence, FBXO32 dysregulation promotes the EMT process in urothelial carcinoma after acquisition of platinum resistance. This was verified by accumulation of MyoD proteins and the subsequent increase in the expression of the mesenchymal markers Snail and vimentin and the reduction in the expression of the epithelial molecule E-cadherin [113]. However, interestingly, Sahu et al. reported conversely that FBXO32 directly targeted CtBP1 for its ubiquitination and enhanced its nuclear retention, which generated a suitable microenvironment for EMT progression and cancer metastasis in human breast cancer cells [114]. Moreover, in the same study, the expression of FBXO32 was elevated in breast cancer cells with invasive properties (MDA-MB-231 cells), and depletion of FBXO32 in a xenograft mouse model suppressed tumor growth and metastasis [114].

FBXO5, which is also known as EMI1 and FBX5, has been identified as an endogenous inhibitor of the anaphase-promoting complex or cyclosome (APC/C) that plays an oncogenic role in human cancers. It has been reported that upregulation of FBXO5 is correlated with increased growth and instability of p53-deficient cells, which suggests that depletion of p53 may have a crucial function in tumorigenesis together with FBXO5 [115]. Previous studies have shown that FBXO45 is an atypical F-box protein induced by estrogens [116]. Further studies have revealed that the FBXO45 protein is responsible for neural development via the ubiquitin-protein ligase E3 [117] and that it plays a pivotal role in controlling synapse formation [118], terminal synaptic growth [119], neurotransmitter release [120], and neural differentiation [118] via ubiquitin-induced proteolysis. In addition, FBXO45 was found to induce the degradation of p73, which belongs to the p53 tumor suppressor family [121]. Furthermore, knockdown of FBXO45 enhances the stability of p73 and promotes cell apoptosis, which indicates that FBXO45 might have an oncogenic function in carcinogenesis [121].

A recent study based on data collected from the TCGA and Gene Expression Omnibus (GEO) databases showed that the expression of FBXO45 and FBXO5 was apparently increased in squamous cell lung cancer (SqCLC) compared with normal lung tissues [122]. Similarly, a prognostic analysis conducted by the K-M plotter database also revealed that overexpression of FBXO45 and FBXO5 contributed to shorter overall survival in patients with SqCLC [122]. Further molecular experiments showed that knockdown of FBXO5 increased cell apoptosis but that knockdown of FBXO45 promoted EMT in SqCLC cells [122]. However, FBXO45 does not form an SCF complex because it associates with the RING finger-type ubiquitin ligase PAM (protein associated with Myc) to produce a novel SKP1-PAM-FBXO45 complex without Cullin1 [118]. Moreover, the SKP1-PAM-FBXO45 complex interacts with Snail1/2, Twist1, and ZEB1/2 through the SPRY domain and F-box domain of FBXO45, which promotes ubiquitination-based degradation of these core EMT transcription factors [123]. Conversely, the level of FBXO45 is negatively regulated by miR-27a, which leads to the inhibition of degradation of these EMT factors by FBXO45 and to EMT progression [123]. FLASH, a caspase-8-associated protein 2 (also known as CASP8AP2), was found to prevent ZEB1 degradation mediated by SIAH1/2 E3 ligases and the SKP1-PAM-FBXO45 atypical ubiquitin E3 ligase complex, thereby leading to an upsurge of EMT [124]. Further mechanistic studies are required to define the physiological role of FBXO45 in carcinogenesis and EMT progression.

Most recently, several studies focused on the role of the FBXO2, FBXO22, and FBXO31 proteins in EMT regulation. FBXO2 is an SCF ubiquitin ligase substrate adaptor that preferentially and specifically binds to high-mannose glycoproteins, and it is highly enriched in the brain [125, 126]. Xu et al. revealed that knockdown of FBXO2 by siRNA reduced the proliferation and metastasis of human gastric cancer cells and inhibited EMT, as evidenced by the upregulation of E-cadherin and the downregulation of N-cadherin and vimentin [127]. Thus, targeting FBXO2 may be a potent therapeutic strategy for gastric cancer patients with higher expression of FBXO2. FBXO22 is a novel, well-characterized F-box protein that mediates the degradation of Lysine (K)-specific demethylase 4A (KDM4A), Kruppel-like factor 4 (KLF4) and methylated p53 [128–130], although the biological role of FBXO22 is still largely undefined. One group demonstrated that FBXO22 exhibited antitumor effects by targeting Snail for ubiquitin-triggered proteasomal degradation, which suppressed the progression of EMT and metastasis in breast cancer [131]. Specifically, a patient-derived W52R mutation within the F-box domain impaired FBXO22-mediated Snail degradation [131]. Previous studies have indicated that FBXO31 is a senescence-related protein, as it targets and degrades Cyclin D1, which plays an important role in G1/S entry [132, 133]. Furthermore, FBXO31 has been defined as a tumor suppressor in various human malignancies such as breast, ovarian, hepatocellular, and prostate cancers, since it targets Cdt1 for degradation in G2 phase of the cell cycle to prevent replication and carcinogenesis [132, 134]. Similarly, further evidence has shown that FBXO31 represses EMT by mediating the ubiquitin-proteasomal degradation of Snail1 in gastric cancer [135]. Since studies that focus on how these FBXO proteins regulate EMT during cancer progression are extremely rare, more studies should be performed to discover novel FBXO proteins that are involved in EMT modulation and to elucidate the molecular mechanism of FBXO-triggered EMT. In summary, FBXO2 promotes EMT, while FBXO11, FBXO45, FBXO22, and FBXO31 inhibit EMT in human cancer cells. Moreover, the role of FBXO32 in EMT regulation is dependent on the cancer type.

## Regulation of cancer stem cells by F-box proteins

Emerging evidence has revealed the critical role of F-box proteins in cancer stem cells. For example, two groups reported that depletion of FBXW7 significantly promotes EMT and the generation of CSCs in colorectal cancer [44] and cholangiocarcinoma cells [136]. Notably, FBXW7 deletion results in stem cell activation and leukemogenesis via upregulation of the Notch target c-Myc [137]. FBXW7 targets Notch activity and thus is also characterized as an essential negative regulator in breast cancer stem cell expansion. Prolyl-isomerase Pin1 sustains Notch signaling by opposing the FBXW7 effects on Notch degradation and promotes breast cancer stem cell self-renewal, tumor growth, and metastasis [138].

Castration-resistant prostate cancer (CRPC) is one of the most common malignant diseases in males due to a lack of effective targeted therapy. Ruan et al. showed that SKP2 stabilized the Twist protein and prevented its ubiquitination and degradation by β-TrCP, which promoted EMT and CSCs, thereby playing a fundamental role in the formation and progression of CRPC [139]. Skp2 positively regulates cancer stem cell populations and CSC self-renewal ability in human cancers, which suggests that targeting Skp2 could restrict cancer stem cells and cancer progression [88]. It has also been reported that Skp2 governs basal homeostasis and stress-induced hematopoietic stem cell regeneration [140, 141]. Similarly, Skp2 is a key regulator of hematopoietic stem cell quiescence, frequency, and self-renewal capability. Depletion of Skp2 enhances hematopoietic stem cell populations via stimulation of cell cycle entry. Notably, depletion of Skp2 enhances sensitivity of leukemia cells to chemotherapy agents, which indicates that Skp2 might be a target for leukemia stem cell treatment [142]. In addition, Skp2 overexpression, which was found to be associated with poor prognosis, maintains the cancer stem cell pool and increases CSC self-renewal ability in nasopharyngeal cancer [143]. NDRG1 (N-myc downstream regulated gene 1) promotes stem-like properties via direct interaction with Skp2 and downregulation of Skp2 phosphorylation in nonsmall cell lung cancer [144].

β-TrCP was also reported to regulate cancer stem cells in various types of human cancers. For example, increased β-TrCP activity targets the REST transcriptional repressor, which leads to the inhibition of cancer stem cell proliferation [145]. SOX9 represses β-TrCP-mediated protein degradation and increases the nuclear GLI1 level and thus promotes cancer stem cell properties [146]. FBXL14 overexpression induces the degradation of c-Myc, promotes glioma stem cell differentiation, and represses tumor growth. In line with this, the deubiquitinase USP13 is highly expressed in glioma stem cells, where it stabilizes c-Myc by antagonizing FBXL14-mediated ubiquitination; this in turn enhances stem cell self-renewal and tumor growth [147]. Ueda et al. reported that Fbxl10 overexpression in murine hematopoietic stem cells contributed to leukemia via upregulation of Nsg2 (neuron-specific gene family member 2) and metabolic activation [148]. FBXL7 and FBXW11 are also involved in UCHL1 (ubiquitin carboxyl-terminal esterase L1)-mediated stem-like cancer cell function in high-grade glioma [149].

## Conclusions and perspectives

**Fig. 1:**
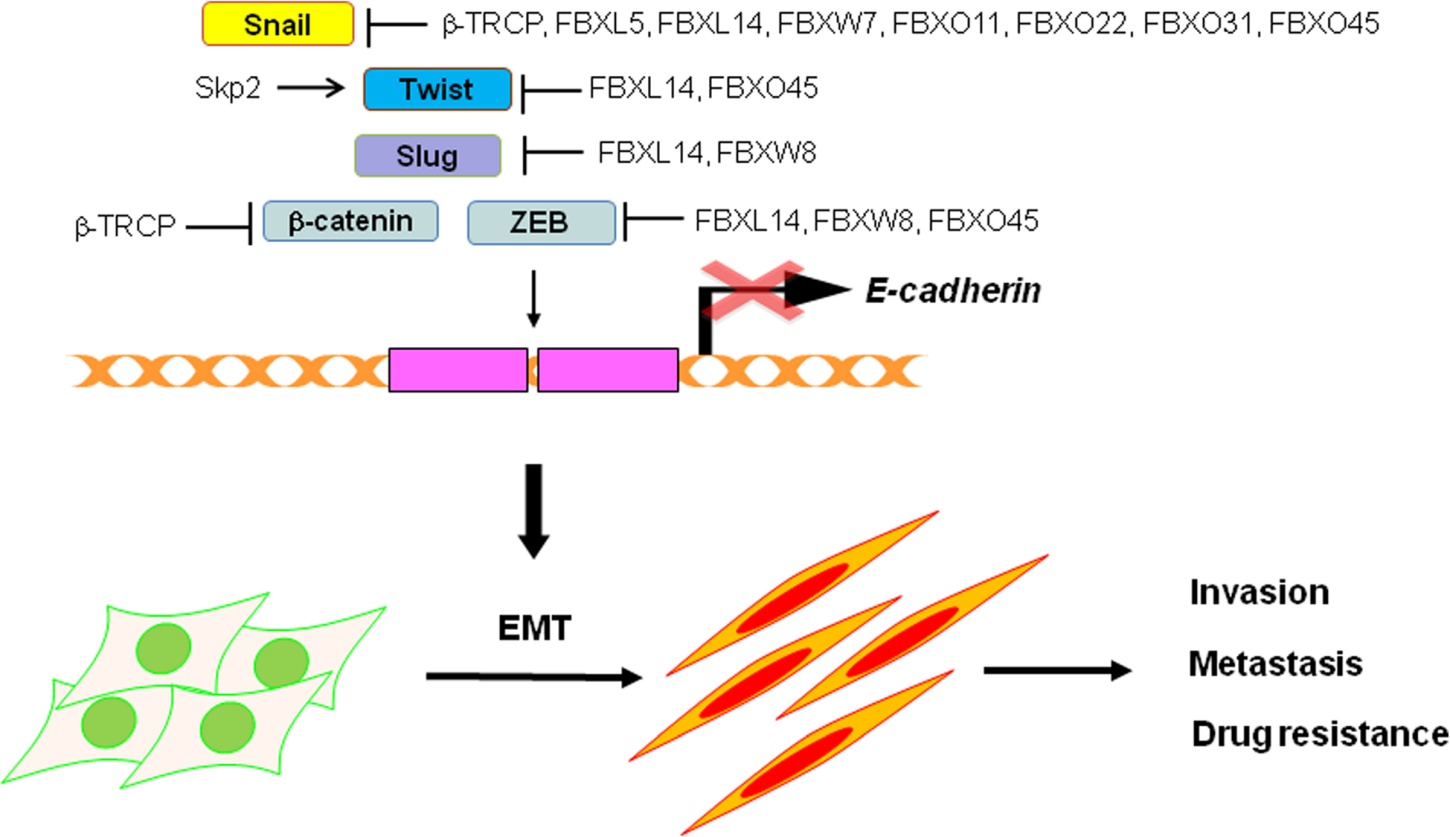
Illustration of how F-box proteins govern EMT-inducing transcription factors. F-box proteins regulate EMT through proteolysis of EMT-inducing factors. FBXL, F-box and leucine-rich repeat protein; FBXW, F-box and WD-40 domain protein; FBXO, F-box only protein

The EMT process plays a crucial role during development, while inappropriate activation of EMT significantly contributes to tumor progression and metastasis. This process is primarily regulated by multiple EMT-related factors including E-cadherin, β-catenin, Snail, Slug, Twist, and ZEB, whose levels and stability could be directly driven by different F-box proteins through targeting and mediating the ubiquitination-based degradation of these EMT factors (Fig. 1). EMT is associated with cancer stem cells and drug resistance. Therefore, in this review, we introduced multiple F-box proteins and discussed their physiological or pathological functions in controlling EMT progression and cancer stem cells. However, many open questions remain and need to be addressed in the future. For example, among the 69 F-box proteins, how many other F-box proteins are involved in EMT and cancer stem cells in human cancers? The detailed molecular mechanisms of how F-box proteins govern the EMT process and cancer stem cells need to be elucidated. Moreover, transgenic mouse models should be used to validate the role of F-box proteins in regulating EMT and cancer stem cells. In addition, it is necessary to determine whether F-box protein expression is associated with EMT and cancer stem cell marker changes in clinical tumor tissues. We believe that these future studies will lead to novel therapeutic strategies that involve targeting F-box proteins in human cancers.

## Abbreviations

APC/C: Anaphase-promoting complex or cyclosome
CASC2: Cancer susceptibility candidate 2
CRL: Cullin-RING E3 ligase
CRPC: Castration-resistant prostate cancer
CSCs: Cancer stem cells
EMT: Epithelial-mesenchymal transition
GEO: Gene Expression Omnibus
GSK-3β: Glycogen synthase kinase-3β
HCC: Hepatocellular carcinoma
HECT: Homologous to E6-associated protein C-terminus
KDM4A: Lysine (K)-specific demethylase 4A
KLF4: Kruppel-like factor 4
lncRNA: Long noncoding RNA
LRR: Leu-rich repeat
MMP: Matrix metalloproteinase
MROC: Methotrexate-resistant osteosarcoma cancer
NDRG1: N-myc downstream regulated gene 1
NF-κB: Nuclear factor-κB
NSCLC: Nonsmall cell lung cancer
RCC: Renal cell carcinoma
RING: Really interesting new gene
SCF: SKP1-cullin 1-F-box protein
SKP1: S-phase kinase-associated protein 1
TCGA: The Cancer Genome Atlas
TGF-β: Transforming growth factor-β
UCHL1: Ubiquitin carboxyl-terminal esterase L1
UPS: Ubiquitin-proteasome system
ZEB: Zinc-finger E-box-binding
β-TrCP: β-Transducin repeat-containing protein
